# O055: Can incidence of surgical site infections (SSI) in hospitals be predicted from point prevalence surveillance data of SSI?

**DOI:** 10.1186/2047-2994-2-S1-O55

**Published:** 2013-06-20

**Authors:** H Jamaladin, JA Ferreira, LD Kuijper, MC Vos, M Koek

**Affiliations:** 1 Epidemiology and Surveillance, RIVM, Bilthoven, The Netherlands; 2 Department of Health Sciences, VUmc, Amsterdam, The Netherlands; 3 Department of Medical Microbiology and Infectious Diseases, Erasmus MC, Rotterdam,The Netherlands

## Introduction

SSIs are one of the most frequent nosocomial infections. To monitor and reduce SSI-rates a good surveillance is crucial. For optimal information, surveillance of incidence of SSIs is preferred above surveillance of prevalence of SSIs. Incidence surveillance however is time consuming.

## Objectives

To investigate whether the prevalence of SSIs (point prevalence surveillance) can be used to adequately predict the incidence of SSIs (cumulative incidence surveillance).

## Methods

Data were derived from the Dutch surveillance network for nosocomial infections (PREZIES) from 2007 to 2011. The suitability of the Rhame and Sudderth method to estimate incidence of SSIs from prevalence of SSIs was assessed. Also incidence data were used to simulate prevalence data, and prediction models were developed to predict incidence from prevalence and from other relevant variables. Several statistical indices were used to evaluate the performances of the models.

## Results

Use of the Rhame and Sudderth method to estimate incidence resulted in most estimated incidence rates becoming negative values (below zero). Simulating prevalence from incidence data showed large variation in prevalence depending on the day of measurement. The predictive model best predicting incidence, with a proportion explained variance of 0.31, was the model including the mean length of hospitalization of patients with an SSI (LN), the mean interval between admission and onset of the SSI (INT) and hospital (as random effect). Adding prevalence to the prediction model did not improve the model.

## Conclusion

It proved not reliable to directly convert prevalence into incidence using the Rhame and Sudderth method. The negative estimated incidence values were the result of the postdischarge surveillance mandatory for the SSI-surveillance in the Dutch surveillance network. Also the simulations and the results of the prediction model indicate that with the current data available it is not possible to accurately predict cumulative incidence of SSIs in Dutch hospitals using point prevalence data.

## Disclosure of interest

None declared.

